# Supplementary material for: New models for donor-recipient matching in lung transplantations
Source: PLoS One. 2021 Jun 4;16(6):e0252148. doi: 10.1371/journal.pone.0252148 (PMC8177410; doi:10.1371/journal.pone.0252148)
Supplement: Attachment — Submitted filename: Response to Reviewers.pdf [file pone.0252148.s004.pdf]

Reviewer's Responses to Questions

Comments to the Author

1. If the authors have adequately addressed your comments raised in a previous round of review and you feel that this manuscript is now acceptable for publication, you may indicate that here to bypass the “Comments to the Author” section, enter your conflict of interest statement in the “Confidential to Editor” section, and submit your "Accept" recommendation.

Reviewer #1: (No Response)

Reviewer #3: All comments have been addressed

---

2. Is the manuscript technically sound, and do the data support the conclusions?
The manuscript must describe a technically sound piece of scientific research with data that supports the conclusions. Experiments must have been conducted rigorously, with appropriate controls, replication, and sample sizes. The conclusions must be drawn appropriately based on the data presented.

Reviewer #1: Yes

Reviewer #3: Yes

---

3. Has the statistical analysis been performed appropriately and rigorously?

Reviewer #1: Yes

Reviewer #3: Yes

---

4. Have the authors made all data underlying the findings in their manuscript fully available?
The PLOS Data policy requires authors to make all data underlying the findings described in their manuscript fully available without restriction, with rare exception (please refer to the Data Availability Statement in the manuscript PDF file). The data should be provided as part of the manuscript or its supporting information, or deposited to a public repository. For example, in addition to summary statistics, the data points behind means, medians and variance measures should be available. If there are restrictions on publicly sharing data—e.g. participant privacy or use of data from a third party—those must be specified.

Reviewer #1: Yes

Reviewer #3: Yes

---

5. Is the manuscript presented in an intelligible fashion and written in standard

English?

PLOS ONE does not copyedit accepted manuscripts, so the language in submitted articles must be clear, correct, and unambiguous. Any typographical or grammatical errors should be corrected at revision, so please note any specific errors here.

Reviewer #1: (No Response)

Reviewer #3: Yes

---

6. Review Comments to the Author
Please use the space provided to explain your answers to the questions above. You may also include additional comments for the author, including concerns about dual publication, research ethics, or publication ethics. (Please upload your review as an attachment if it exceeds 20,000 characters)

Reviewer #1: This revision clearly has changes in it but I cannot tell without a copy of track changes. Also, in your responses, there is no point by point response, just a thank you for the comments. This is very sparse. It used to be a COVID lung transpalnt paper, and now its just an analysis of the Spanish registry. Please submit a trach changes so I can tell what is new. If not, then since this has been changed from a "COVID" paper to this, please submit a new submission.

As indicated, at the request of the reviewers we have omitted the reference we made specifically in this COVID-19 pandemic to the benefits of applying machine learning, artificial intelligence, to the lung donor and recipient allocation process of using artificial intelligence. We have tried following your directions to seek general benefits in the lung donation and transplantation program based on the ability of the machines to learn independently and make accurate predictions. We not only treat a Spanish registry analysis, but rather we try to apply artificial intelligence combined with the “big data” of electronic medical records to achieve the highest precision when matching donor-recipient organs and optimize our results.

Regarding changes and modifications made in the article as you request me attached:

A marked copy of the manuscript that highlights the changes made to the original version. It appears uploaded as a separate file labeled "Revised Manuscript with Track Changes." 'Revised Manuscript with Track Changes'.

An unmarked version of the revised document with no recorded changes. Uploaded this as a separate file labeled 'Handwritten'. 'Manuscript'.

Reviewer #3: Dear The Authors

Thank you for writing this manuscript
I have couple of points to mention here:

1. Please run a second round of English language spell check for the whole manuscript

We have requested the English spelling check of the manuscript from the team of translators at the University of Córdoba (UCO). I hope it is greatly improved in this version.

2. Please remove the part pertaining to COVID-19 since your study period is covering before the pandemic.

We update the text without specifying benefits of applying artificial intelligence and machine learning specifically in the COVID-19 pandemic to generalize to all aspects of the Donation and Lung Transplant process.

Thank you

---

7. PLOS authors have the option to publish the peer review history of their article (what does this mean?). If published, this will include your full peer review and any attached files.

Reviewer #1: No

Reviewer #3: **Yes:** salah eldien Altarabsheh
