# Supplementary material for: New models for donor-recipient matching in lung transplantations
Source: PLoS One. 2021 Jun 4;16(6):e0252148. doi: 10.1371/journal.pone.0252148 (PMC8177410; doi:10.1371/journal.pone.0252148)
Supplement: Attachment — Submitted filename: Response to Reviewers.doc [file pone.0252148.s003.doc]

Reviewer #1: I commend the authors on their fine work. With COVID, the allocation of lungs is even more difficult.

Please review for grammar and english syntax. There are multiple grammatically incorrect sentences in the introduction. It seems very disjointed and I cannot go over the whole thing in this review. Also I think the authors meant for incomplete sentences to be opening sentences of paragraphs. but they just appear as incomplete sentences.

Grammar and syntax has been reviewed by professionals from the language translation team at the Maimonides Biomedical Research Institute of Córdoba. I hope this time your understanding is more appropriate.

From reading the introduction, it seems like the authors are using COVID as a buzz word. Besides the fact that COVID decreased transplants, how else is it affecting lung transplantation. How have institutions dealt with this? There should be more regarding COVID and transplantation if the authors want to bring up COVID.

Initially, the work was designed prior to the appearance of COVID, although we think it could be interesting to include it to give originality to our work, we recognize its inclusion may be somewhat artificial at this time.

In accordance with your comments on the appearance of COVID terms, and also following the recommendations of our other reviewer, we have removed everything related to COVID from our text.

As for the methods section, I have asked the editors to have a statistician review the statistics as I am not well versed in neural netorks.

Our computer science and numerical analysis team will be happy to collaborate in solving any questions about the statistics and development of neural networks

With the results of the study, please draw some conclusions. The conclusion section is basically a restatement of all the results, which is inappropriate for a conclusion. Also, if the authors would like to discuss COVID and transplant, please review this in the conclusion. No where in the conclustions is there anything about COVID.

Work and activities aimed at improving the problem of the shortage of organs for transplantation, should include measures to optimize donors suffering brain death, to promote live donation, evaluate expanded criteria for organ transplantation, and increase donation in

asystole or with the used of ex vivo lung recovery machines. We believe that the model we obtained in this retrospective review of the donor-recipient lung allocation system may be a new way forward for optimizing the lung donation and transplantation process. This system should also consider additional variables of transplantation benefit, i.e., allocation considering survival and maximized use given the increased demand for organs which is encouraging the transplantation of organs by applying expanded criteria.

Artificial neural networks, based on data and variables resulting from the experience and results of multiple transplants, could play an important role in matching lung transplant donor-recipients, thus optimizing efficiency, effectiveness, and equity, and helping to avoid any subjectivity that may play a personal role in decision-making by professionals. The fusion of LR and neural networks can provide information on the importance of each of the non-linear relationships between variables that influence the evolution of lung transplantation. Finally, it would be interesting to validate these results, both internally and externally, and to carry out a prospective multicenter clinical trial validation to try to achieve greater robustness when applying the allocation model in lung the context of transplantation allocations.

Reviewer #2: This isnq retrospective review on f a Spanish lung transplant registry data which is evaluated for new paradigms

First and foremost you often state that this is a problem relative to COVID which it is not. The data are from before Jan 2016 well before any COVID infection and the data is never applied t patients undergoing transplant during the COVID era. Please remove all references to COVID in the title and most other places

Initially, the work was designed prior to the appearance of COVID, although we think it could be interesting to include it to give originality to our work, we recognize its inclusion may be somewhat artificial at this time.

In accordance with your comments on the appearance of COVID terms, and also following the recommendations of our other reviewer, we have removed everything related to COVID from our text.

What about perfusion circuits and their effect on the variable of cold ischemia?

When talking about perfusion circuits and cold ischemia, I understand you are referring to donation in asystole. The data that appear in our work refer to donor cases in brain death. In our center, the asystole donation program began until 2013, the number of cases being too low to be included in the study. In addition, we believe it could include some confounding factor due to its characteristics.

Regarding the exvivo pulmonary perfusion system, although the results are being very satisfactory in terms of improving cold ischemia results, our center does not have enough experience to include them in the study.

You imply that this will help with organ shortage? How would that happen!?l

This work would not reduce the problem of organ shortage, rather it would help to optimize the distribution of organs between donor and recipient so that they lost the fewest number of grafts after transplantation.

The results indicate the high complexity of the problem treated and the need to introduce other variables in this classic analysis. The accuracy of the model is promising. and it would be able to optimize the principles of justice, efficiency and equity.

With the application of this model, complications such as primary graft dysfunction, infections, rejection and many others present in lung transplant patients would be minimized. These decisions would favor that our grafts were more durable and therefore the life of our recipients was longer, ensuring the success of the donation and transplantation process.

Did yu plan a multicenter preferably international validation study?

In the future, external validation of the system would be interesting to know if the neural network is capable of predicting the result of transplantation in a population other than the Spanish one. It will probably be necessary to start this validation with data similar to those selected in this work and progressively improve the neural networks to be able to incorporate data related to different races, other types of transplants such as asystole, and increase the prevalence of underlying diseases or different etiologies as can be the case of paracetamol poisoning or diseases autoimmune.

English good ammar needs upgrading
